# Feeding Problems in Typically Developing Young Children, a Population-Based Study

**DOI:** 10.3390/children8050388

**Published:** 2021-05-13

**Authors:** Katerina Sdravou, Maria Fotoulaki, Elpida Emmanouilidou-Fotoulaki, Elias Andreoulakis, Giorgos Makris, Fotini Sotiriadou, Athanasia Printza

**Affiliations:** 1 4th Department of Pediatrics, School of Medicine, Aristotle University of Thessaloniki, General “Papageorgiou” Hospital, 56403 Thessaloniki, Greece; sdravouk@tcd.ie (K.S.); mfotoul@otenet.gr (M.F.); elpisemfot@hotmail.com (E.E.-F.); fotinisotiriadou@gmail.com (F.S.); 2 Adult Psychiatric Unit, Hellenic Centre for Mental Health and Research, Department of Thessaloniki, 36 Kaftatzoglou Str., 55337 Thessaloniki, Greece; eliasandreoulakis@gmail.com; 3 Department of Speech and Language Therapy, School of Health Sciences, University of Peloponnese, 2400 Kalamata, Greece; glog_makris@yahoo.gr; 4 1st Otolaryngology Department, School of Medicine, Aristotle University of Thessaloniki, University Hospital AHEPA, 54636 Thessaloniki, Greece

**Keywords:** feeding problems, prevalence, food intake, typical development, healthy children, food neophobia, consumption of vegetables

## Abstract

Feeding problems have been estimated to occur in approximately 25–45% of normally developing children. The aim of this study was to investigate the prevalence of feeding problems in typically developing young children in Greece. Child feeding behavior, parents’ feelings about their child’s feeding patterns, and parental feeding practices were also explored. Parents completed the Greek version of the Behavioral Pediatrics Feeding Assessment Scale (BPFAS). Data on 742 healthy, typically developing children aged two to seven years are presented. Overall, the majority of children in the sample showed high frequency of desirable mealtime behaviors and low frequency of undesirable mealtime behaviors. However, a significant proportion of the cohort presented with food neophobia and low consumption of vegetables. When applying test cut-off scores, it was found that 8.2% of the sample had abnormal Total Frequency Score (TFS) and 26.6% had abnormal Total Problem Score (TPS). The study showed that parent-reported feeding problems are quite common in children of typical development in Greece. Moreover, while the majority of the sample displayed a high frequency of favorable behaviors, specific child feeding behaviors are amenable to improvement.

## 1. Introduction

Feeding development occurs without difficulties in most of the typically developing children. However, feeding problems in early childhood are quite common and are of great concern both to parents and pediatricians worldwide [1,2,3]. Feeding problems range from mild to severe [1,2] and are associated with a number of negative effects on organic, psychological, developmental, and social aspects which vary from mild (e.g., missed meals) to severe (e.g., failure to thrive) [4,5,6]. Feeding problems encompass a variety of heterogeneous problems whose development and maintenance involve many different factors. This fact makes their classification complicated and as a result many researchers give different definitions of feeding problems such as “feeding problem”, “food refusal”, “selective eating”, “food selectivity”, “picky eating”, “fussy eating”, and “dietary restriction”. Other researchers identify three basic categories of children who exhibit unfavorable feeding behavior; children with limited appetite, children with selective intake, and children with fear of eating [1,2].

A combination of clinical observation, interview and questionnaire use is commonly applied to assess feeding problems in children [7]. Estimation of the prevalence on a large geographical scale, however, is not feasible by clinical evaluation. Therefore, the use of validated questionnaires is required, providing the collection of measurable and comparable information. Estimates of the prevalence of feeding problems in typically developing children have considerable deviation in the literature, attributed both to discrepancies in the assessment methods and to definition issues (Table 1) [8,9,10,11,12,13,14,15,16,17,18,19,20,21,22,23,24,25,26]. Most of the existing studies used sets of questions to assess feeding problems whereas only one has used a reliable questionnaire that reported a “fussy” eating behavior profile in 5.8% of 4-year-olds [25]. In fact, most studies are focusing on the estimation of selectivity (picky eating) prevalence, but even in this case, there are deviations regarding not only the measurement methods, but also the definition of selectivity [8]. A recent systematic review ascertains that the high heterogeneity in the definition of selectivity among studies resulted in an estimated prevalence of selectivity ranging from 5.8% to 59% [9]. Indicative of these extensive discrepancies is a study in which different prevalence values were estimated for the same children sample with the use of different criteria [10]. More specifically, 25% of the parents stated that their child was selective whereas 47% of the same sample met the criteria of “selectivity” and 15% met the criteria of “feeding problems” when feeding behaviors were assessed with multiple questions [10]. It is also worth noting that some of the questions that “distinguished” selectivity from other feeding problems concerned behaviors that constitute a feeding problem themselves (e.g., “eating slowly or holding food in the mouth”). Thus, variance in the prevalence of feeding problems may be related to caregiver difficulty in identifying specific types of feeding problems, or differences in the use of definitions across methods of measurement when structured approaches are used. Moreover, variance in the prevalence of feeding problems among the prevalence studies may also be related to the different and broad age range of the participants of each study. Developmentally typical feeding behavior and problems are different at varying ages; therefore, inconsistent prevalence results may be reported (e.g., neophobia in toddlers decreases with age).

In the latest version of the DSM-5 manual [27], a new diagnostic term, “Avoidant/Restrictive Food Intake Disorder” (ARFID) was introduced. ARFID is recognized as an eating disorder which manifests with selectivity, decreased appetite, or food phobia. It is characterized by persistent failure of the individual to meet adequate nutritional and/ or energy needs, being manifested by one (or more) of the following: “weight loss or inability to gain weight”, “significant nutritional deficiencies”, “dependence on dietary supplements or enteral feeding” and “significant impairment of psychosocial function” [27,28,29,30,31]. However, children whose primary challenge is a skill deficit are excluded from the diagnosis of ARFID. Regarding ARFID, there is currently a very limited number of epidemiological studies. According to a recent systematic review based on 16 studies, prevalence estimates range from 1.5% to 64% in clinical groups with eating disorders [32]. Among these, only three concerned a healthy population and the prevalence reported ranged from 0.3% to 15.5%. More specifically, the prevalence of ARFID in people older than 15 years of age in Australia was 0.3% [33]. Ιn Portugal, 15.5% of children aged 5 to 10 possibly experienced ARFID. To assess the disorder, parents were given a five-question questionnaire concerning the basic symptoms of ARFID; decreased interest in food, selectivity, insufficient growth, weight loss or inability to gain weight, and dependence on enteral feeding or dietary supplements [34]. A national epidemiological study of psychiatric diseases in children aged 7 to 14 years in Taiwan reported a low prevalence of ARFID (<1%) [35].

Moreover, the International Statistical Classification of Diseases and Related Health Problems, 10th Revision (ICD-10) diagnostic codes includes two codes to identify pediatric feeding problems. More specifically, F98.2 (Other feeding disorders of infancy and childhood) requires the absence of organic disease whereas the inadequately defined R63.3 (Feeding difficulties) is used as an umbrella term covering a broad range of disorders [36,37]. Addressing this need for a specific, well defined diagnostic term and in order to adequately describe the multiple aspects of a feeding problem, Goday et al. proposed the use of the International Classification of Functioning, Disability, and Health (ICF) framework to define a unifying diagnostic term, “Pediatric Feeding Disorder” (PFD) [37]. PFD refers to age-inappropriate impairment of oral intake, but also takes into consideration consequent dysfunction in at least one of four closely related, complementary domains (medical, nutritional, feeding skills, and psychosocial). This definition illustrates the multifaceted aspects of PFD highlighting the physiologic and functional impact of the disorders. Moreover, adoption of this definition provides a common terminology that is necessary in clinical practice and research and enables a multidisciplinary approach to the patients.

The aforementioned findings make explicit that conclusions regarding estimates of prevalence of feeding problems in healthy pediatric population cannot be safely drawn. This is mainly due to the great diversity both in the definition of feeding problems and in the use of different outcome measures as well as the methodological weaknesses of prevalence studies, most of which used outcome measures based on experience rather than valid and reliable psychometric tools for assessing or detecting feeding problems. Conclusions about the prevalence of moderate to severe feeding problems (e.g., meeting criteria for a diagnosis such as PFD or ARFID) that interfere with nutritional intake and require specialized intervention to address are more difficult to be drawn. This is attributed to the inefficiency of parent-reporting questionnaire scores to justify or constitute a clinical diagnosis of a feeding disorder. Validated questionnaires are indeed useful in the diagnostic process, but clinical interview and observation from a trained professional is required by any standard.

To date, the Behavioral Pediatrics Feeding Assessment Scale (BPFAS) [38] appears to have the highest comprehensive reliability and validity data among parent-administered feeding questionnaires for preschool children [38]. Content, construct, and concurrent validity data are also available for the specific questionnaire [38,39,40,41]. The questionnaire has good test–retest reliability [39,42] and internal consistency [43,44,45], acceptable to good sensitivity, and good to excellent specificity [42,46]. The BPFAS is capable of capturing a wide range of feeding difficulties (nutritional and textural selectivity, food refusal, and oral motor difficulties). Furthermore, it is one of the few questionnaires for which discrete diagnostic cut-off points have been estimated which can be used to detect feeding problems [46]. To the best of our knowledge, however, it has not been used to date in a feeding problem prevalence study. There is a lack of epidemiological studies on feeding problems in Greece. In this context, the present study aims to provide an estimation of the prevalence of feeding problems in typically developing young Greek children using the BPFAS. Moreover, child feeding behavior and parental feeding practices as well as parents’ feelings about their child’s feeding patterns and mealtimes at home are also explored.

## 2. Material and Methods

This cross-sectional study was approved by the Bioethics and Ethics Committee of the Medical School at Aristotle University of Thessaloniki as well as by the Greek Ministry of Education. Written informed consent was obtained from all parents who participated in the study.

### 2.1. Participants

A sample representative of children of typical development, aged 2–7 years was recruited from public and private preschools (kindergartens) from all geographical regions in Greece. After being informed by the primary investigator for the purposes of the study, 75 out of 121 contacted kindergartens accepted to participate to the study. The parents of 742 children (one parent for each child) participated in the study. Inclusion criteria comprised typically developing children aged 2–7 years and parents with Greek as their mother tongue. Exclusion criteria included a medical history of developmental, neurological disorders, gastrointestinal, or other chronic diseases that could affect feeding habits or weight gain, a medical history that might affect the motor pattern of swallowing, e.g., prematurity and a history of swallowing problems. The criteria related to the medical history could not be examined prior to addressing the parents; therefore, they were assessed retrospectively (after the return of the questionnaires) and respective cases were excluded according to the parent’s report of medical issues.

A hard copy of the questionnaires was either mailed or personally handed to the school principals by the principal investigator. The school principals distributed the information leaflet, the parent consent form, and the questionnaires to the parents. Parents wishing to participate returned the completed questionnaires to the principals. The parents filled out the BPFAS questionnaire, the parent and child demographics form, and a child’s medical history questionnaire. The sample collection lasted from November 2016 until June 2018.

### 2.2. Measures

#### 2.2.1. Demographic and Anthropometric Data

The parents’ demographic characteristics such as sex, age, educational level, and employment status alongside the children’s demographic data such as sex, age, presence of siblings, and birth order were recorded. Moreover, anthropometric measures including birth weight, current height, and weight were also registered. The World Health Organization’s Anthro [47] and AnthroPlus [48] softwares were used in order to convert height and weight scores to age- and gender-specific BMI z-scores.

#### 2.2.2. Child’s Medical History

The child’s medical history was recorded via 13 questions. Most of the questions were of “yes–no” type while there were some multiple-choice and two open-ended questions (birth weight and gestational age). Regarding the content of the questions, there were four about the perinatal history (e.g., birth weight, type of delivery), two about the presence of feeding problems, and three referring to disorders or diseases that may affect feeding or are common in children with feeding problems (e.g., chronic diseases, gastrointestinal disorders). One question concerned the presence of speech or attention deficit disorder and three were related to weight gain during the first year of life, after the first year of life, and during the last three months.

#### 2.2.3. Behavioral Pediatrics Feeding Assessment Scale (BPFAS)

BPFAS has the highest reliability and validity in the detection of feeding problems among a wide variety of available psychometric tools aiming to detect feeding problems. Its validity has been tested in different populations and in various age groups. BPFAS has been proven to have sufficient sensitivity to evaluate therapeutic interventions [39,41,42,45,49,50,51,52]. Concerning its structure, the scale has two sections and 35 questions in total. The first 25 questions constitute the first section and evaluate the child’s feeding behavior, while the remaining 10 questions comprise the second section and assess the parents’ feelings about their child’s feeding patterns and mealtimes at home and the strategies they adapt to deal with potential problems. For each one of the 35 questions, parents were requested to answer how often a named behavior is observed (on a five-point scale from 1-never to 5-always) and if the specific behavior is a problem for them (yes–no). This results in two different scores, the Total Frequency Score (TFS) (maximum score 175) and the Total Problem Score (TPS) (maximum score 35), respectively. Individuals with scores higher than 84 for the TFS and 9 for the TPS are considered to be at risk of feeding problems. The Greek-version BPFAS has been used in the present study [53].

## 3. Statistical Analysis

Summary statistics for all variables of interest were calculated, as appropriate, i.e., mean and standard deviation were provided for scale variables such as child’s age, zBMI, birthweight, TFS, and TPS, whereas absolute (Number, N) and relative (percentage, %) frequency were calculated for the categorical variables such as the demographics, the answers to each of the 35 items of BPFAS scale, and the class where each participant would fall according to TFS and/or TPS cut-off. A Kolmogorov–Smirnoff test was performed so as to test whether scale variables (TFS and TPS) were normally distributed or not. Then, in order to investigate for any association between the demographics and/or anthopometrics of the participants and the possibility of a feeding problem (the latter indicated by abnormal TFS and/or TPS), chi-square tests or Student’s *t*-tests were performed for categorical (demographics) and scale variables (child’s age, zBMI, and birthweight), respectively. Finally, Pearson’s r correlation coefficient was calculated in order to test the correlation between raw TFS and TPS (regardless of whether they exceeded the relevant cut-off or not) with the aforementioned scale variables of the study. In the cases where the normality assumptions would not hold, non-parametric tests (i.e., Mann Whitney’s U test and Spearman’s Rho correlation coefficient) would be used, accordingly. The level of statistical significance (alpha) was set at 0.05. SPSS Statistics v20 statistical software (IBM, 142, Armonk, NY, USA) was used for the analysis.

## 4. Results

The parents of 742 typically developing children participated in the study. The two sexes were equally represented for children (372 girls and 370 boys). The mean children’s age was 4.92 ± 1.00 years with 341 (46.0%) of the children being 5 years old or younger. Firstborns represented the 52.2% of the sample, whereas 22.8% of the children had no siblings. The mean birthweight of the children was 3268.43 ± 444.80 g, whereas current zBMI was 0.28 ± 1.41. The vast majority of participating parents were mothers (92.6%). Almost three quarters of the parents (73.9%) were aged below 40 years and 45% of them had secondary education (12 years) or lower. The relevant results are presented in Table 2.

The mean TFS was 62.71 ± 14.23 and the mean TPS was 6.23 ± 6.40. Given the established cut-offs which are indicative of feeding problems, the prevalence of feeding problems was 8.2% (according to TFS) and 26.6% (according to TPS). The relevant results are presented in Table 2.

Attempting to describe the main characteristics of the feeding profile of the children in this study, one could summarize that the consumption rates for various foods (i.e., the percentage of children consuming a certain food “very often” or “always”) were as follows: 80.8% for starches, 80.2% for meat or fish, 74.8% for milk, 60.6% for fruits, and 52.9% for vegetables. Certain unfavorable feeding behaviors were prevalent (with a frequency of “very often” or “always”) as follows: food neophobia (will not try new foods): 29.8%; decreased appetite: 10.9%; prolonged mealtimes (meal duration greater than 20 min): 36.7%; and negotiation over eating (what to eat): 31.8%. However, 77.1% of the children came readily to mealtime and 79.8% enjoyed eating according to parental reports.

As for the parents, 32.2% stated that they were not confident that their child got enough to eat, 27.4% admitted that they did not feel confident that they could manage their child’s behavior at mealtime, whereas 6.5% reported frustration or anxiety when feeding their child. A considerable percentage of the parents were found to succumb to their negative feelings about their child’s feeding patterns and employ questionable practices in order to get their child to eat. Some of the frequently used practices (“very often” to “always”) were cooking something else (12.9%), coaxing (7.7%), threatening (2.7%), or even physically forced feeding (1.9%).

Investigating associations, the correlation coefficient between the TFS and TPS was found to be 0.713 (*p* < 0.001), revealing a strong correlation. TFS and TPS were negatively correlated with zBMI (Spearman’s rho −0.131; *p* = 0.001 and −0.106; *p* = 0.006, respectively) whereas TPS was positively associated with the child’s age (Spearman’s rho 0.083; *p* = 0.026). The relevant results are presented in Table 3.

Associations between pathological TFS or TPS (i.e., values above the established cut-offs) and demographics were examined. It was found that sex was not associated with pathological TFS or TPS. The same was true for parental educational and employment level. On the contrary, being the firstborn or having a parent aged below 40 was associated with a pathological score in both TFS and TPS. There were some factors such as being the only child, and being older than 5 years that were associated with pathological TPS but not TFS. The same was true (a higher possibility for a pathological TPS but not TFS score) when the father was the informant instead of the mother. The last finding, however, has to be interpreted with caution, since fathers were underrepresented in the study. The relevant results are presented in Table 4.

## 5. Discussion

Although feeding problems are very common in young children, conclusions regarding estimates of their prevalence in typically developing children cannot be safely drawn. This may be attributed to definition issues along with discrepancies in the assessment methods used in previous studies. The present study employs the BPFAS aiming to investigate the prevalence of feeding problems in typically developing young children in Greece. Regarding the frequency of problematic feeding behaviors, the mean TFS of the present study (62.71 ± 14.23) is similar to that found in a previous study in the Greek population (62 ± 15, 100 participants) [53] and comparable to a Canadian (63.9 ± 14.2, 96 participants) [49] and an English cohort study (62.50, 509 participants) [46]. Moreover, it is lower than a typical sample of Australia (TFS = 68.1 ± 15.7, 54 participants) [40] and higher than that reported for a US sample (TFS = 54.3, 89 participants) (*p* < 0.001) [45]. Regarding the number of behaviors perceived as problematic by parents (TPS), the mean TPS of the present study (6.23 ± 6.40) is consistent with a previous study on a typical Greek sample (6.2 ± 6.3) [53]. However, it is higher than that of an Australian (4.1 ± 6.2) [42], American (2.8) [43], Canadian (3.0 ± 4.5) [49], and English (TPS = 2.7) sample [46]. This might indicate that Greek parents are probably more inclined to report problematic feeding behaviors compared to populations in other countries (at least in the West, for which data are available). This finding is possibly attributed to socio-cultural differences and may reflect differences in the beliefs and expectations of Greek parents on feeding their children. These differences may have important implications on feeding practices of Greek parents and consequently on how they influence their children’s eating behavior. Indeed, previous research demonstrated that a large percentage of Greek parents had significant control over how much their child eats and often disregarded their child’s satiety [54,55].

When the established cut-offs were used, it was found that 8.2% and 26.6% of the sample had abnormal TFS and TPS, respectively. According to previous studies, the estimated prevalence of feeding problems ranged from 4.8% to 52%, with the majority of them reporting prevalence around 10% or around 20–30% [10,11,12,13,14,15,16,17,18,19,20,21,22,23,24,25,26]. Both estimated prevalence rates (via TFS and TPS) in the present study are in accordance with the above-mentioned studies. The deviation of the percentages estimated via TFS and TPS probably indicates a differentiation of the two scores on detecting the “severity” of the feeding problem. Thus, abnormal TFS may be indicative of more severe feeding difficulties, while abnormal TPS may be more sensitive in detecting even the milder feeding problems. Milano et al. suggested that 25–50% [1,3,8,21,56] of typically developing young children experience feeding difficulties whereas only 10% of them face feeding difficulties that are severe enough in order to require systematic intervention [1]. Indeed, the two different proportions of the present study (8.2% based on TFS and 26.6% based on TPS) appear to be consistent with this estimation, supporting the aforementioned interpretation. Another possible explanation is that while the TFS score concerns the record of a child’s behavior, the TPS score illustrates the parent’s evaluative judgment over this behavior (whether it is considered a problem for them or not). The observed deviation between the two scores could therefore also represent the distance that sometimes exists between the description of reality and its subjective experience and interpretation. In this regard, we could conclude that while 26.6% of parents consider their child’s feeding behavior problematic, only about one third of these children (i.e., 8.2%) seem to present significant feeding problems.

Almost one third of the parents in our study were concerned about the reduced consumption of vegetables. The findings demonstrate that almost half of the children in the sample did not consume vegetables often enough, while the same was ascertained for about 40% of children regarding fruit consumption. The findings of the present study are supplementary to the already existing literature confirming the alarmingly reduced consumption of fruits and vegetables by children. This phenomenon seems to be global, as it is reported that, in general, the average intake of fruits and vegetables is lower than the recommendations of the World Health Organization (WHO) [57], with the issue of vegetable consumption particularly concerning. For example, just over one third of school-age children in Europe consume vegetable on a daily basis [58].

Another issue that often appeared to concern parents in the present study was food neophobia, which was severe in about 30% of the children. This finding is in accordance with most of the studies on food neophobia that report prevalence around 30% [59]. However, estimates of food neophobia prevalence vary widely in the international literature (from 12.8% to 100%) [59]. This high variability is due to the diversity of tools used to measure food neophobia. Although the causes of food neophobia have not been fully elucidated, it is considered to be related to a number of biological, psychological, and environmental factors, most notably the children’s innate predisposition to sweet and savory tastes, the influence of food sensory characteristics, the time and method of introducing new foods, parental practices (e.g., coercive practices, lack of motivation), child familiarization with different tastes, and the temperament characteristics of the child (e.g., increased stress levels) [59,60] Although food neophobia manifests at the age of 2 to 5 years and is usually eliminated later, the severity of this behavior significantly affects the way a child eats and can affect a person’s eating habits in later life [60]. These effects are mainly related to the lack of a balanced diet and reduced important nutrient intake.

The high prevalence of feeding problems in the present study highlights the importance of early detection and treatment of feeding problems among young children. This is very important, taking into account the fact that most caregivers do not ask for professional help, probably because they consider feeding problems as a typical part of growing up [61]. Research evidence suggests that many feeding problems are preventable or easily treated but, if left untreated, feeding problems may lead to severe complications. A multidisciplinary approach is usually required to prevent the potential impact of feeding problems, improve growth, nutritional status, and quality of life and guarantee feeding safety [62]. Considerable evidence supports the use of various treatment options such as behavioral approaches (e.g., implementation of mealtime structure, appetite manipulation, reinforcement-based procedures, systematic desensitization or graduated exposure, food play, and parent training [63,64,65,66,67]) and methods that improve feeding skills (e.g., oral motor exercises). Moreover, the high prevalence of both selectivity regarding fruits and vegetables and the severe degree of food neophobia demonstrated in the present study indicate the need for nutritional education in young children in preschool (e.g., kindergarten, nursery school) as well as in the family environment. It is important to inform parents about positive practices that contribute to the improvement of the child’s diet quality and to adjust these practices according to the child’s age.

Similar to Canadian [49], US [43], and UK [46] normative groups, the age or sex were not related to the frequency of feeding problems for the Greek normative sample either. However, the age and age group (being older than 5 years) were strongly related to pathological TPS in the present study. This finding suggests that parents of older children more often considered their child’s feeding behavior to be problematic (TPS) although the actual frequency of problematic behaviors was not increased (TFS). Therefore, we can assume that behaviors that, at a younger age, were not perceived as a problem (probably because they were considered expected for the child’s developmental age), when they manifested or persisted at an older age, were considered a problem by the parents. This finding emphasizes the significant importance of early detection of problematic behaviors, which usually occur between six months and four years [68], and their prompt treatment.

Furthermore, being the first born was related to feeding problems in the present study. This finding is consistent with previous studies in which first-born children were more likely to develop feeding problems [21,69], whereas Crist and Napier-Phillips [49] did not find a similar correlation. In addition, being the only child was related to pathological TPS but not TFS. Previous studies on Greek population have shown that being firstborn and being an only child had a significant effect on the environment and on parental feeding practices, which may justify the findings of the present study. Another possible explanation may lie in the fact that firstborns and only children are deprived of the benefit of imitating the feeding behavior of their older siblings (peer modeling).

Parental age (aged < 40 years) was also associated with feeding problems in the current study indicating the need for appropriate counseling especially for younger parents. Benjasuwantep [21] reported that maternal or paternal age did not differ between healthy children with or without feeding disorders. Furthermore, a cross-sectional study [70] found no association between maternal age and low food variety or low drive to eat. Similar results are reported by the cross-sectional study of Hendricks [71] which investigated the role of maternal age in multiple aspects of feeding practices, but there was no association between maternal age and food neophobia. In contrast, Cassells’ study [72] supported that maternal age was inversely related to food neophobia in the multiple regression analyses although initial analyses of potential covariates revealed a low positive correlation between maternal age and food neophobia. Another interesting finding of our study is the association of mothers with abnormal TPS. This suggests a sex-oriented assessment of the child’s feeding behavior with a possible underestimation of the condition by the fathers or, in contrast, overestimation by the mothers. However, given the small percentage of fathers who participated in the study, no safe conclusions can be drawn and this hypothesis needs to be verified by larger studies.

TFS and TPS were negatively correlated with zBMI and abnormal TFS and TPS were associated with lower zBMI. The design of the present study (cross-sectional) does not allow inferring causal relationships. Feeding problems may be therefore due to low BMI or conversely, feeding problems may be the cause of low BMI. This is particularly important, highlighting the nutritional impact of feeding problems that, if left untreated, may lead to nutritional deficiencies. Nutritional deficiencies are also a key element for the diagnosis of ARFID. PFDs might also be associated with nutritional complications [37]. Results of previous research are contradictory, with several studies not finding any association between feeding behavior and nutrition [39,45,73,74]. Many of these, however, involved clinical groups that possibly achieved to maintain the desirable level of nutrition under special nutritional support. A recent systematic review [9] on specific feeding problems (food neophobia and picky eating) highlighted that previous relevant results were disparate. In particular, the study could not determine a clear association between food neophobia and weight status, and regarding picky eating, the relevant results were conflicting, ranging from no association to association with either underweight or overweight status, possibly attributed to inconsistencies in the definition and measurement of picky eating.

The aim of this study was to investigate the prevalence of feeding problems in typically developing young children. An important strength of the study is the use of a reliable parent-administered feeding questionnaire. In addition, the present study used a large sample size which was collected from a vast geographic range including urban and rural areas. As far as we know this is the first study that used the estimated cut-offs of BPFAS to assess feeding problems in typically developing children and it is the first prevalence study on feeding problems in Greece.

A limitation of our study, unavoidable in all studies that use self-completing questionnaires compared to clinical trials, concerns the reliability of these scales and possible over- or underestimation errors. On the other hand, the objective evaluation of feeding problems through clinical examination is extremely difficult and challenging to carry out on a large scale. For these reasons and because the aims of the present study required a large and representative sample, BPFAS was used, which is considered a more objective clinical measurement tool to detect feeding problems. However, prevalence levels, although in agreement with previous studies of similar methodology, should be treated with caution as clinical evaluation is necessary in order to confirm the diagnosis of a feeding problem. Moreover, the voluntary participation of parents in the study may itself introduce a selection bias, as parents who are more actively involved in feeding their child or experience feeding difficulties may be more inclined to participate. Therefore, they form a sample where feeding problems are relatively common. As a consequence, the estimated prevalence of feeding problems in the present study may possibly be an overestimation of feeding problems that actually exist in the general population. Furthermore, the generalization of the present findings to other populations should be attempted with caution, as eating behavior is significantly influenced and largely shaped by the wider social context in which it manifests. Finally, this study is purely descriptive, thus it presents correlation data. As such, it remains unclear how many of these problems develop over time. In addition, there are no treatment data. Thus, it is unclear if the BPFAS would be successful in identifying changes in feeding problems and especially when it is used as a pre/post measure of feeding problems in young children. Thus, future studies are necessary in order to investigate two aspects of BPFAS, the ability of the questionnaire to discriminate clinical from normative samples and the sensitivity to display changes after therapeutic intervention in the Greek population.

## 6. Conclusions

Given the high prevalence of feeding problems in young children, further epidemiological studies using reliable and valid diagnostics tools are essential. Our results suggest that about one in ten children seem to display feeding problems as reported by parents, and one in four children are considered by their parent to have a significant problem. The findings of the study highlight the need to improve awareness and early detection of feeding difficulties in children of typical development where feeding problems are often overlooked or underestimated.

## Figures and Tables

**Table 1:** Main findings of feeding problem prevalence studies *.

Study	Outcome Measure of Feeding Problem	Population (Participants, Age)	Country	Prevalence
Marchi and Cohen, 1990 [11]	Interview with mothers	800 children 1–10 years old	USA	29%
Rydell et al., 1995 [12]	Set of questions **	240 children 6.1–11.0 years old	Sweden	30%
Reau et al., 1996 [13]	Set of questions	130 infants 151 toddlers 13–27 months	USA	33% of infants 52% of toddlers
Cerro et al., 2002 [14]	Set of questions	95 children aged 1.5–3.5 years	New Zealand	20%
Jacobi et al., 2003 [15]	Single question	135 children 3.5–5.5 years	USA	21%
Esparo et al., 2004 [16]	Set of questions	851 children 3–6 years	Spain	4.8%
Dubois et al., 2007 [17]	Set of questions	1498 preschoolers, 2.5, 3.5, and 4.5 years old	Canada	14–17%
Wright et al., 2007 [18]	Set of questions	455 parents 30 months	UK	8.3%
Hittner and Faith, 2011 [19]	Set of questions	487 children 1 and 3 years old	USA	9%
Micali et al., 2011 [20]	Set of questions	1327 children 5 to 7 years old	Denmark	7.3% picky eating 1.4% poor eating
Goh et al., 2012 [10]	Set of questions	407 parents/grandparents of children aged 1 to 10 years	Singapore	25.1% picky eating 15.2% difficulties
Benjasuwantep et al., 2013 [21]	Set of questions	402 children Aged 1–4 years	Thailand	26.9%
Dubois et al., 2013 [22]	Set of questions	692 (346 twin siblings) 2.5 and 9 years old	Canada	9.4% at 2.5 years 10.7% at 9 years
Hafstad et al., 2013 [23]	Set of questions	913 children 1.5 to 4.5 years old	Norway	22–35%
Equit et al., 2013 [24]	Set of questions	1090 children 4–7 years old	Germany	23.2% picky eating 4.8% food avoidance
Tharner et al., 2014 [25]	Child Eating Behavior Questionnaire (CEBQ)	3117 children Aged 4 years old	The Netherlands	5.8%
Haszardet al., 2015 [26]	Set of questions	203 overweight children 4–8 years	New Zealand	36.5%

* All studies refer to a general description of feeding problems (including more than one unfavorable feeding behaviors such as food refusal, picky or fussy eating, prolonged mealtimes, lack of appetite etc.) and not to a particular behavior (e.g., picky eating); ** purpose made set of questions for the specific research.

**Table 2:** Demographic and anthropometric characteristics of the sample and BPFAS scores.

		*Ν* (%)
Child sex	Female	372 (50.1)
Child age group	>5 years	401 (54.0)
Only child	yes	169 (22.8)
Firstborn	yes	387 (52.2)
Parental sex	Female	687 (92.6)
Parental age group	<40 years	548 (73.9)
Parental education	>12 years	408 (55.0)
Working parent	yes	529 (71.3)
TFS score by cut-off	>84	61 (8.2)
TPS score by cut-off	>9	189 (26.6)
Child’s age (years)	Mean ± SD Median (Q1, Q3)	4.92 ± 1.00 5.17 (4.42, 5.67)
BMI z-score (current)	Mean ± SD Median (Q1, Q3)	0.28 ± 1.41 0.24 (−0.57, 0.24)
Birth weight (grams)	Mean ± SD Median (Q1, Q3)	3268.43 ± 444.80 3230 (3000, 3550)
TFS score	Mean ± SD Median (Q1, Q3)	62.71 ± 14.23 60.00 (52.75, 70.00)
TPS score	Mean ± SD Median (Q1, Q3)	6.23 ± 6.40 4.00 (1.00, 10.00)

**Table 3:** Bivariate correlations between TFS and TPS score and age, zBMI, and birthweight.

	TFS Score	TPS Score
TFS		**0.713 (<0.001)**
TPS	**0.713 (<0.001)**	
Child age	0.053 (0.150)	**0.083 (0.026)**
zBMI	**−** **0.131 (0.001)**	**−** **0.106 (0.006)**
Birth Weight	−0.048 (0.191)	−0.030 (0.417)

Spearman’s rho correlation coefficient (*p* value). Significant correlations are shown in bold.

**Table 4:** Comparison of children with high vs. low TFS/TPS score in relation to demographics.

		TFS				TPS			
		TFS ≤ 84 *Ν* = 681 *Ν* (%)	TFS > 84 *Ν* = 61 *Ν* (%)	Chi Square	*p*	TPS ≤ 9 *Ν* = 522 *Ν* (%)	TPS > 9 *Ν* = 189 *Ν* (%)	Chi Square	*p*
Child sex	Female	341 (50.1)	31 (50.8)	0.012	0.911	268 (51.3)	87 (46.0)	1.565	0.211
Child age group	>5 years	364 (53.5)	37 (60.7)	1.170	0.279	272 (52.1)	117 (61.9)	5376	**0.020**
Only child	Yes	150 (22.0)	19 (31.1)	2.648	0.104	107 (20.5)	54 (28.6)	5.163	**0.023**
Firstborn	Yes	342 (50.2)	45 (73.8)	12.443	**<0.001**	255 (48.9)	116 (61.4)	8.724	**0.003**
Parent sex	Female	629 (92.4)	58 (95.1)	0.603	0.438	475 (91.0)	181 (95.8)	4.425	**0.035**
Parent age group	<40 years	496 (72.8)	52 (85.2)	4.467	**0.035**	370 (70.9)	153 (81.0)	7.236	**0.007**
Parental education	Higher	373 (54.8)	35 (57.4)	0.153	0.695	282 (54.0)	109 (57.7)	0.746	0.388
Working parent	Yes	485 (71.2)	44 (72.1)	0.023	0.880	374 (71.6)	132 (69.8)	0.221	0.639
		TFS ≤ 84 Mean ± SD	TFS > 84 Mean ± SD	U test (z)	*p*	TPS < 9 Mean ± SD	TPS ≥ 9 Mean ± SD	U test (z)	*p*
Child age				U = 22,533.0 z = 1.100	0.272			U = 55,351.0 z = 2.490	**0.013**
zBMI		4.92 ± 0.99	4.98 ± 1.09	U = 13,848.0 z = −3.210	**0.001**	4.88 ± 1.00	5.07 ± 0.95	U = 37,233 z = −2.570	**0.010**
Birth weight		0.33 ± 1.36	−0.25 ± 1.76	U = 23,744.0 z = 1.854	0.064	0.34 ± 1.36	0.07 ± 1.46	U = 47,928.5 z = −0.579	0.563

Note: Percentages refer to the columns, i.e., within the classes of TFS or TPS score and not to the lines (demographic variable). Significance is shown in bold.
